# GPU-accelerated deformation mapping in hybrid organ models for real-time simulation

**DOI:** 10.1007/s11548-025-03377-2

**Published:** 2025-07-07

**Authors:** Rintaro Miyazaki, Yuichiro Hayashi, Masahiro Oda, Kensaku Mori

**Affiliations:** 1 Graduate School of Informatics, Nagoya University, Furo-cho, Chikusa-ku, Nagoya, 464-8601 Japan; 2 Information Technology Center, Nagoya University, Furo-cho, Chikusa-ku, Nagoya, 464-8601 Japan; 3 Research Center for Medical Bigdata, National Institute of Informatics, Hitotsubashi, Chiyoda-ku, Tokyo, 101-8430 Japan

**Keywords:** Deformation, Surgical simulation, Octree, GPU, Vertex shader

## Abstract

**Purpose:**

Surgical simulation is expected to be an effective way for physicians and medical students to learn surgical skills. To achieve real-time deformation of soft tissues with high visual quality, multiple resolution and adaptive mesh refinement models have been introduced. However, those models require additional processing time to map the deformation results of the deformed lattice to a polygon model. In this study, we propose a method to accelerate this process using vertex shaders on GPU and investigate its performance.

**Methods:**

A hierarchical octree cube structure is generated from a high-resolution organ polygon model. The entire organ model is divided into pieces according to the cube structure. In a simulation, vertex coordinates of the organ model pieces are obtained by trilinear interpolation of the cube’s 8 vertex coordinates. This process is described in a shader program, and organ model vertices are processed in the rendering pipeline for acceleration.

**Results:**

For a constant number of processing cubes, the CPU-based processing time increased linearly with the total number of organ model vertices, and the GPU-based time was nearly constant. On the other hand, for a constant number of model vertices, the GPU-based time increased linearly with the number of surface cubes. These linearities determine a condition that the GPU-based implementation is faster in the same frame time.

**Conclusion:**

We implemented octree cube deformation mapping using vertex shaders and confirmed its performance. The experimental results showed that the GPU can accelerate the mapping process in high-resolution organ models with a large number of vertices.

## Introduction

Laparoscopic surgery is performed using long and thin surgical instruments with an endoscopic camera that inserted through one or more small incisions in the abdomen. Unlike traditional open surgery, it is a minimally invasive operation because the holes made in the patient are small. However, the restricted field view displayed on a 2D monitor through an endoscopic camera causes a lack of depth perception for the surgeon. Therefore, surgeons need to learn the techniques for laparoscopic surgery before the actual operation on the patient. Simulation-based surgical training is expected to be utilized due to its ethical and economical advantages over the use of cadavers or animals. In recent years, various simulation-based training systems for laparoscopic surgery have been developed actively by many academic organizations and private companies. Several studies show the effectiveness of learning surgical skills in the simulation environment [1, 2].

To develop a surgical simulator, it is necessary to realize all of the following features in real-time processing: simulation of organ model deformation, interaction between the surgical instruments and organ models, and tactile force feedback from the surgical instruments to the operator’s hands. In particular, the simulation of organ model deformation has not yet achieved a practical resolution even with today’s improved processing performance of PCs. High-resolution organ models that can be processed in real time are desired.

Recent deformation simulations of organ models use “hybrid models,” which independently handle models for deformation calculation, models for detecting collisions between organs and surgical instruments, and high-resolution organ models for rendering. In models for deformation calculation, various methods have been proposed to apply adaptive mesh refinement (AMR) models, which make only the parts with large-deformation high resolution. Some frameworks have also been proposed to handle such hybrid models integrally [3, 4].

While the hybrid model improves visual quality, it requires additional processing to map the deformation results of the deformed lattice to the polygon model. This process must be performed on all polygon vertices of the organ model, which means that high-resolution models are more difficult to process in real time if performed on a CPU.

To solve the problem, we propose a method to accelerate this additional mapping process using vertex shaders. In this study, we employ an octree cube, which is typical of AMR as the lattice structure for deformation calculation. An entire organ model for rendering is divided into pieces according to the cube structure. In a simulation, the vertex coordinates of the organ model pieces are obtained by trilinear interpolation of the cube’s 8 vertex coordinates. This process is described in a shader program and is included in the rendering process executed by the GPU, freeing the CPU from its processing. We implement this method in the Unity, which is a typical development environment.

## Related works

### Hybrid models

Models for representing deformable organs require multiple functions, including deformation calculation, for drawing, and collision processing. When real-time processing is a prerequisite, such as in surgical simulations, it is difficult to increase the resolution of the model for deformation calculation due to the high computational cost of physical simulations. In contrast, models for rendering can be processed fast by GPUs, so it is possible to achieve high resolution. In addition, since the collision processing is a combination of all the number of collision judgment targets, it is also important to try to reduce them.

Khan et al. [5] proposed an intelligent model for cutting simulation to update the mesh topology by deleting intersected triangles, re-triangulation, and refinement. This model is effective for representing cutting operations using a high-resolution mesh model for rendering. However, it is not easy to achieve real-time processing by using this model for rendering as the mesh for deformation calculation. In addition, the model for deformation calculation should be filled inside the organ.

Therefore, tetrahedral or hexahedral grid models are often used for deformation calculations, independent of the rendering model. Pan et al. [6] proposed a hybrid model for cuttable flexible tissue models, consisting of a surface mesh for rendering and a tetrahedral mesh generated from the surface mesh for deformation calculation. The use of hybrid models is thus becoming essential, and it is easy to integrate and handle them in the SOFA framework [3] and the NVIDIA FleX [4], which are commonly used for medical simulations.

The advantage of using the deformation calculation model independently from the rendering model is that the vertices of the deformation calculation lattice can be placed independently from the polygon vertices for rendering, and AMR, which will be discussed in the next section, can be easily realized. On the other hand, the disadvantage is that additional computation time is required to map the deformation results of the deformation calculation lattice onto the polygon model. We discuss this in Sect. “Deformation mapping”.

### AMR models

Parallel processing with GPGPUs has also been attempted for real-time processing of deformation calculations. For example, the SOFA framework [3] and the NVIDIA FleX [4] enable easy use of parallelization with GPUs and add-ons. However, the computational complexity of elastic discrete models of 3D objects increases three-dimensionally with its resolution. Therefore, even if parallelization is performed using GPUs, as long as there is an upper limit to the number of cores, the limit of real-time processing cannot be avoided.

Therefore, simply reducing the resolution of the deformation calculation model relative to the resolution of the rendering model is not sufficient for real-time processing. Also, a high resolution is desired for the deformation calculation model in areas with large deformation. Thus, the AMR model, which locally changes the resolution of the deformation lattice, is expected to be adopted. Although AMR is not suitable for parallel processing on GPUs, significant speed-up effects can be expected even in CPU-based processing if the number of processing elements can be greatly reduced.

Tetrahedral or cubic lattice is used for AMR. Tetrahedral lattices have been used in the reference [5, 6]. The advantages of using tetrahedral lattices are that the tetrahedron is a primary element and can share some of its vertices with the rendering model. In contrast, the cubic lattice AMR, octree, directly corresponds to the Cartesian coordinate system and has the advantage of simplifying the implementation. For example, Seiler M. et al. adopted an adaptive octree-based approach and developed interactive cutting of deformable objects [7]. We are also trying local refinement of the octree cube structure around a grasping position [8].

### Shape matching in PBD method

Position-based dynamics (PBD) is a method of physical simulation in computer graphics proposed by Muller et al. [9]. Because of its simplicity and stability, it is often used to generate motion for organ or soft tissue models in surgical simulation.

Fast and stable models of organ deformation are often based on shape matching [10] and PBD methods proposed by Muller et al. To cope with large deformations, it is necessary to consider a rigid body rotation component. In the mass-spring model or PBD method, using the constraint on the distance between the points, the implementation and physical calculation algorithms are simple and efficient. However, there is a fatal problem that overshoot can easily occur during large deformations, and some countermeasures are needed. The shape-based resilience model is one solution to this problem. We applied cube shape matching as a constraint in the PBD method in the previous work [11].

### Deformation mapping

As mentioned above, mapping the result of lattice deformation to the rendering model is required if the deformation calculation model is used independently from the rendering model. This processing time increases with the resolution of the rendering model.

Yvonne et al. [12] proposed a fast method for generating deformations of cube-based models using the NVIDIA CUDA. However, it is not easy to implement this method for GPGPU processing targeting aligned arrays because the resolution changes locally in the octree.

## Methods

This section briefly describes the octree-based deformation model driven by the PBD method, which is the basis of this study [11]. Then, a method is proposed for mapping octree deformation results to a high-resolution organ model on a GPU.

### Overview of octree deformable model

Among spatial partitioning methods with multiple resolutions, octree is easier to handle than other partitioning methods because it fits directly into the Cartesian coordinate system. By devising the arrangement of cubes of multiple sizes, simulations of organ model deformations can be generated with high resolution and high speed. The deformation of an organ model is generated along the following steps.

*Step*1: Overlay an octree model on the target organ model.
*Step*2: Generate the deformation of the octree model.
*Step*3: Map the octree deformation to the organ model vertices.
*Step*4: The organ model is rendered.
*Step*5: Repeat Steps 2 to 4.

### Octree cube layout

**Fig. 1:**
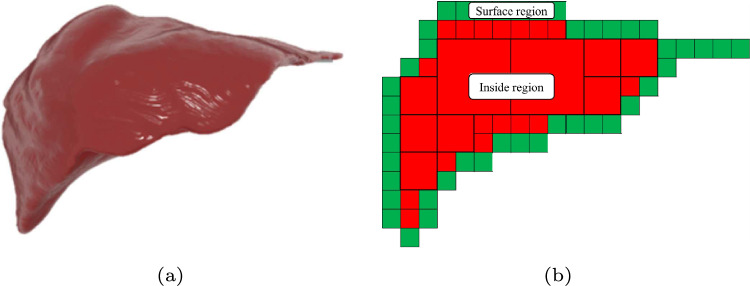
**a** A surface organ model and **b** a sample layout of cube placement. An octree model is generated from a high-resolution organ surface model. Hierarchal cubes are classified into two regions, inside or surface

In Step 1, an octree cube model is generated from the target organ model by method [11]. Figure 1 shows a 2D illustration of a sample of an octree cube layout. As shown in Fig. 1b, each cube is classified into two regions, inside or surface. The cubes of the surface region have vertices of the organ model, while the cubes of the inside region haven’t.

In Step 2, the motion of the octree deformation model is generated by applying the PBD method [9, 13]. This process is applied to all cubes. We use the undeformed state of each cube in the octree as a constraint for the PBD method [11].

**Fig. 2:**
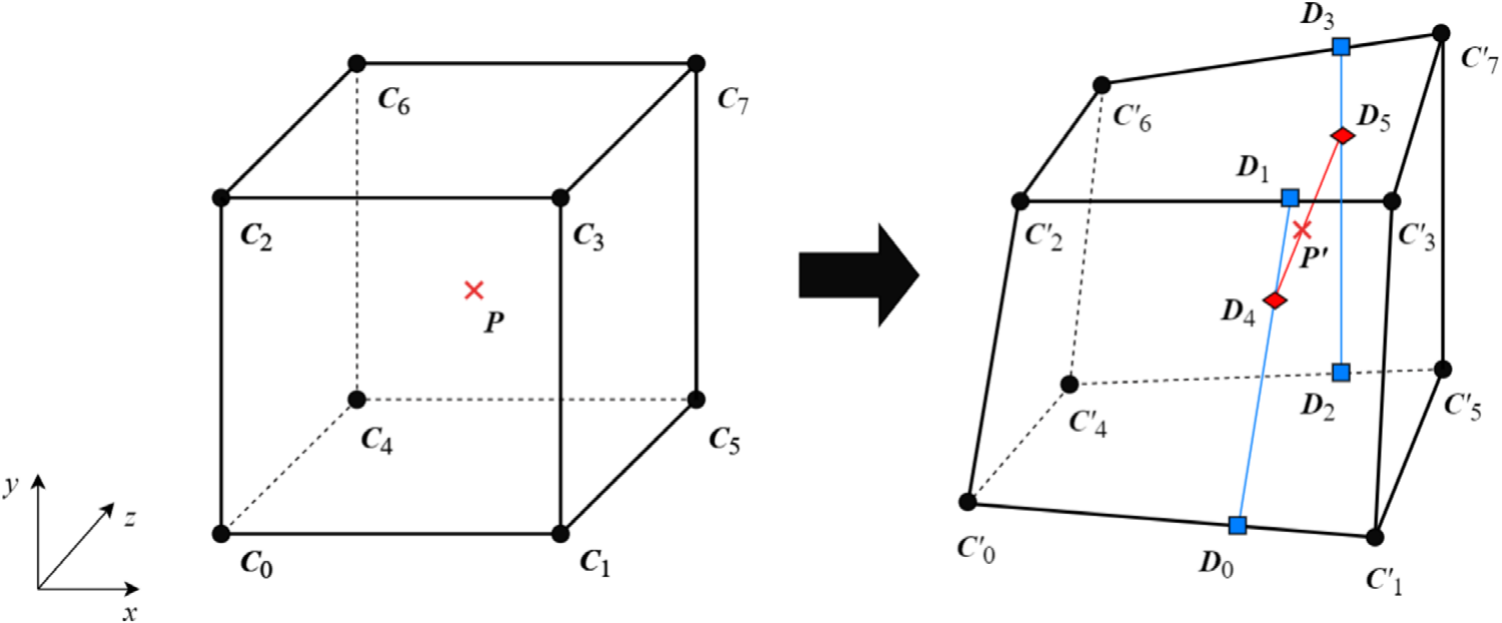
$\varvec{P}'$ is the position $\varvec{P}$ moved due to the deformation, which is obtained from 8-cube vertex coordinates by trilinear interpolation

### Cube deformation mapping

Steps 2 to 4 are performed once per video frame interval. The mapping process in Step 3 is also seen in free-form deformation (FFD), where the movement of control points is reflected in the model to be deformed. This process is done by trilinear interpolation of the 8 vertex coordinates of the cube. This means that the vertex position vector of the organ model is given as input, and the new position vector is obtained along with the deformation of the cube. This process is only performed for cubes classified as surface region, while the PBD method was applied to all cubes in Step 2. The details are described below.

Figure 2 shows that any position $\varvec{P}$ in the cube moves according to the deformation of the cube. $\varvec{C}_i$ and $\varvec{C}'_i\ (i=0, 1, \ldots , 7)$ are the vertices of the cube before and after deformation, respectively. $\varvec{P}'$ is the position of $\varvec{P}$ after the deformation. For trilinear interpolation, the local coordinates $\varvec{P}_{\textrm{Local}}(x, y, z)$ of $\varvec{P}$ in the cube are prepared by normalizing by the length *L* of the cube’s edges. For correct shading, the trilinear interpolation is also applied to the normal vector of each vertex.

1 $$\begin{aligned} \varvec{P}_{\textrm{Local}}(x, y, z) =\dfrac{\varvec{P} - \varvec{C}_0}{L}. \end{aligned}$$

Then, the interior points $\varvec{D}_0$ to $\varvec{D}_5$ are

2 $$\begin{aligned} \varvec{D}_0&= x (\varvec{C}'_1 - \varvec{C}'_0) + \varvec{C}'_0, \end{aligned}$$

3 $$\begin{aligned} \varvec{D}_1&= x (\varvec{C}'_3 - \varvec{C}'_2) + \varvec{C}'_2, \end{aligned}$$

4 $$\begin{aligned} \varvec{D}_2&= x (\varvec{C}'_5 - \varvec{C}'_4) + \varvec{C}'_4, \end{aligned}$$

5 $$\begin{aligned} \varvec{D}_3&= x (\varvec{C}'_7 - \varvec{C}'_6) + \varvec{C}'_6, \end{aligned}$$

6 $$\begin{aligned} \varvec{D}_4&= y (\varvec{D}_1-\varvec{D}_0) + \varvec{D}_0, \end{aligned}$$

7 $$\begin{aligned} \textrm{and} \ \ \varvec{D}_5&= y (\varvec{D}_3-\varvec{D}_2) + \varvec{D}_2. \end{aligned}$$

Finally, $\varvec{P}'$ is obtained as:

8 $$\begin{aligned} \varvec{P}' = z(\varvec{D}_5-\varvec{D}_4) + \varvec{D}_4. \end{aligned}$$

### GPU-based trilinear interpolation

**Fig. 3:**
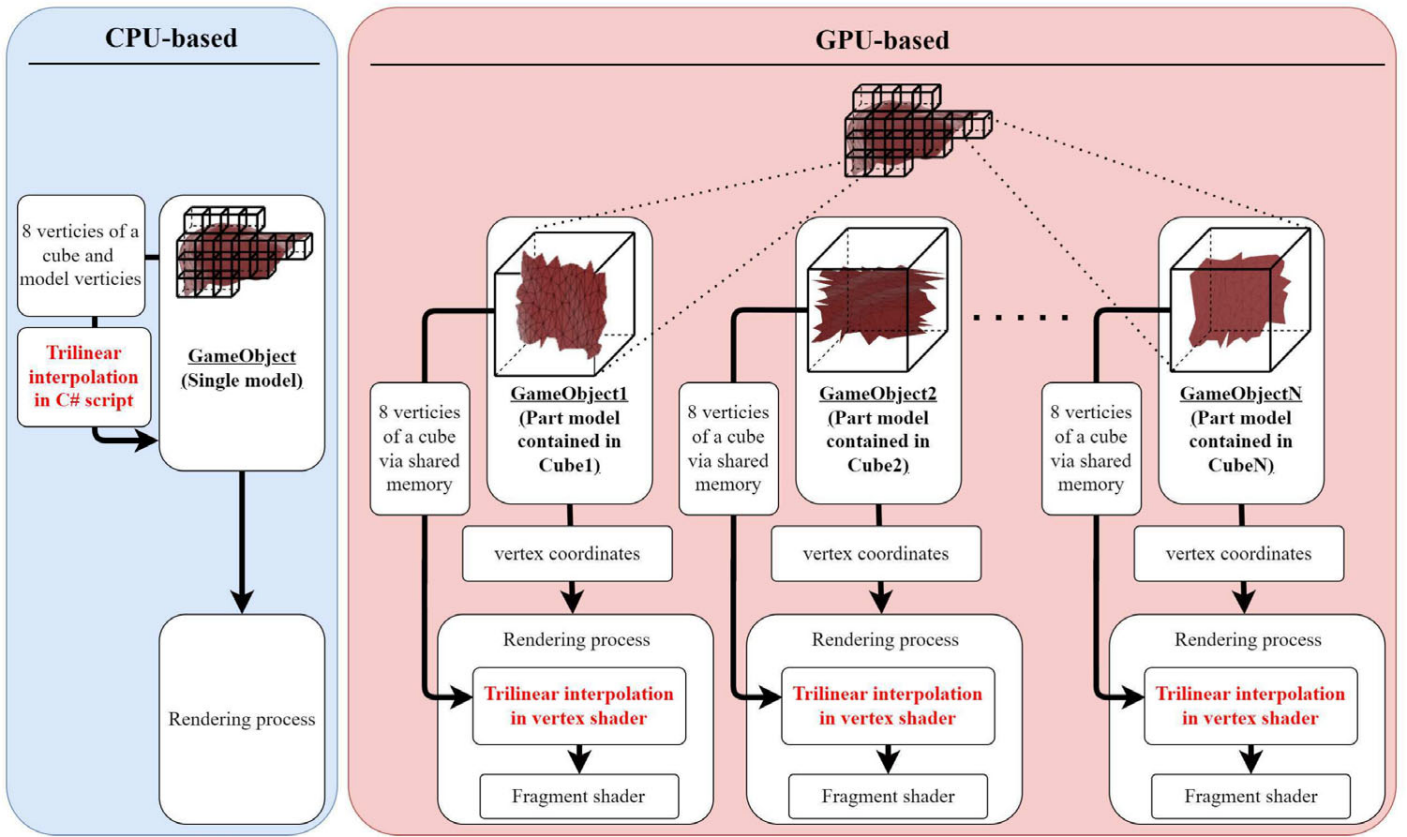
Difference of implementation of trilinear interpolation between CPU and GPU

In the PBD method, the iterative process of motion generation is performed in Step 2, and the number of iterations should be as many as possible to enable real-time processing. However, the mapping process in Step 3 consumes non-negligible processing time, although it is sufficient to perform it once per video frame interval. For example, 16 treads of parallel processing on an AMD Ryzen 3700X, Step 3, take 13 ms for a liver model consisting of approximately 257,000 vertices, which is about 40% of the 33-ms frame interval time at 30 fps. Therefore, we attempt to execute the trilinear interpolation of Step 3 on the GPU. Figure 3 compares the CPU and GPU implementations of the trilinear interpolation process.

In the Unity, the current standard platform for surgical simulation development, a single organ model is treated as a single object, called GameObject. For CPU-based processing, trilinear interpolation is written in a single C# script, allowing easy access to both the vertices of the cube and the organ model.

However, to achieve this with GPU shader processing, the organ model cannot be processed as a single GameObject because the cube vertex coordinates required for trilinear interpolation vary depending on the cube containing each vertex of the organ model. Therefore, the entire organ model is split into multiple objects according to the octree cube arrangement so that a portion of the organ model fits into a single cube, and the 8-cube vertex coordinates used for trilinear interpolation are unique for a single GameObject. Each GameObject has a C#script attached to it to pass the cube vertex coordinates to the vertex shader. The coordinate values of the vertices that are poured into the vertex shader are not absolute 3D positions, but normalized local coordinates in the cube defined by Eq. (1). Within the rendering process, trilinear interpolation is applied to the arriving vertices by the vertex shader process and passed to the fragment shader, the next process in rendering.

## Experiments and results

To verify the effectiveness of vertex shader processing, typical CPU-based and GPU-based methods are implemented. The performance of each implementation was compared in the following two experiments.

*Experiment*1 :  Perform comparison relative to the number of organ model vertices.
*Experiment*2 :  Perform comparison relative to the number of divided GameObjects.

Experiments 1 and 2 were conducted on Unity 2022.3.x LTS platform using a PC with an AMD Ryzen 7 3700X 8-Core 16-Threads Processor 3.60 GHz CPU and an NVIDIA GeForce RTX 3060 GPU. A Universal Render Pipeline (URP) custom shader of Unity was used for shader programming.

Except for shaders, scripts are written in C# following the Unity platform’s scripting environment. While the performance of C# may be lower than that of other low-level programming languages, such as C++, the use of C# is a practical choice in terms of development efficiency. Additionally, the Unity platform internally performs a script conversion called IL2CPP, which reduces performance loss.

As our focus is on the differences in the deformation mapping process in Step 3, we set the number of iterations at 30 in the iterative solver to ensure that the processing time in Step 2 does not affect the results. The other parameters are set within a reasonable range to simulate the movement of the organ model as it falls and collides with a tray.

The processing times for Step 2 (common to both the CPU-based and GPU-based implementations) and Step 3 (specific to the CPU-based implementation) were measured for multi-core processing using the C# “Parallel.For” function, which is provided as a standard C# function. These processing times are referred to as $t_{\textrm{2}}$ and $t_{\textrm{3c}}$, respectively. Measurements were taken using Unity’s “StopWatch” class, averaging of 1000 frames. The remaining times in the CPU-based and GPU-based implementations were denoted as $t_{\textrm{4c}}$ and $t_{\textrm{4g}}$, respectively. These values were calculated by subtracting from the total frame times, $T_{\textrm{c}}$ and $T_{\textrm{g}}$, as

9 $$\begin{aligned} t_{\textrm{4c}}&=T_{\textrm{c}} - t_{\textrm{2}} - t_{\textrm{3c}}, \end{aligned}$$

10 $$\begin{aligned} \textrm{and}\ t_{\textrm{4g}}&=T_{\textrm{g}} - t_{\textrm{2}}. \end{aligned}$$

Note that in the GPU-based implementation, Step 3 of the deformation mapping process is included in $t_{\textrm{4g}}$.

A liver surface model was created by taking segmentation and polygonization of the liver region of the CT volume data. The number of vertices in the surface mesh, referred to as $n_{\textrm{v}}$, was reduced to generate four models containing 39,042, 81,482, 160,052, and 256,904 vertices. These meshes are labeled as Mesh 1 to 4, respectively.

Additionally, we prepared four cases with different cube resolutions in Step 1. Table 1 shows a breakdown of the number of cubes for the cases. The cube levels indicate variations in octree cube sizes, where Level 0 corresponds to the largest cubes and Level 2 to the smallest. The numbers in parentheses represent the number of cubes in the surface region, denoted as $n_{\textrm{c}}$ (see Fig. 1b). This value also corresponds to the number of divided GameObjects.

**Table 1:** Breakdown of number of cubes in octree cube structures

	Case 1	Case 2	Case 3	Case 4
Cube level 0	0	0	4	27
Cube level 1	8	27	85	278
Cube level 2	727 (576)	1207 (929)	2539 (1817)	5318 (3715)
Total	735 (576)	1234 (929)	2628 (1817)	5623 (3715)

The number in parentheses is the number of cubes in the surface region in Fig. 1b

**Table 2:** Processing time values measured for four meshes in Experiment 1

	$n_{\textrm{v}}$	$t_{\textrm{2}}$ [ms]	CPU-based			GPU-based	
			$t_{\textrm{3c}}$ [ms]	$t_{\textrm{4c}}$ [ms]	$T_{\textrm{c}}$ [ms]	$t_{\textrm{4g}}$ [ms]	$T_{\textrm{g}}$ [ms]
Mesh 1	39,042	20.9	2.1	5.1	28.1	10.6	31.5
Mesh 2	81,842	20.9	4.0	5.2	30.1	10.6	31.5
Mesh 3	160,052	20.9	8.2	6.4	35.5	10.6	31.5
Mesh 4	256,904	20.9	13.1	5.7	39.7	10.6	31.5

**Fig. 4:**
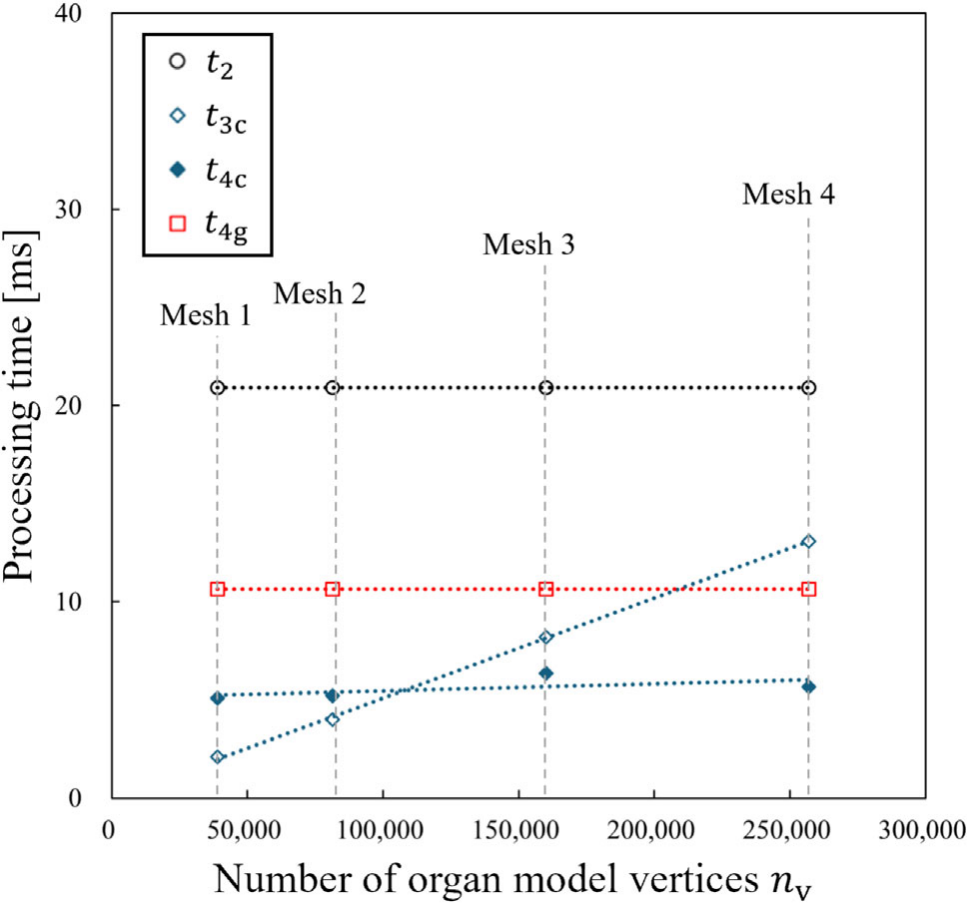
Variation of processing times with respect to $n_{\textrm{v}}$ in Experiment 1

**Fig. 5:**
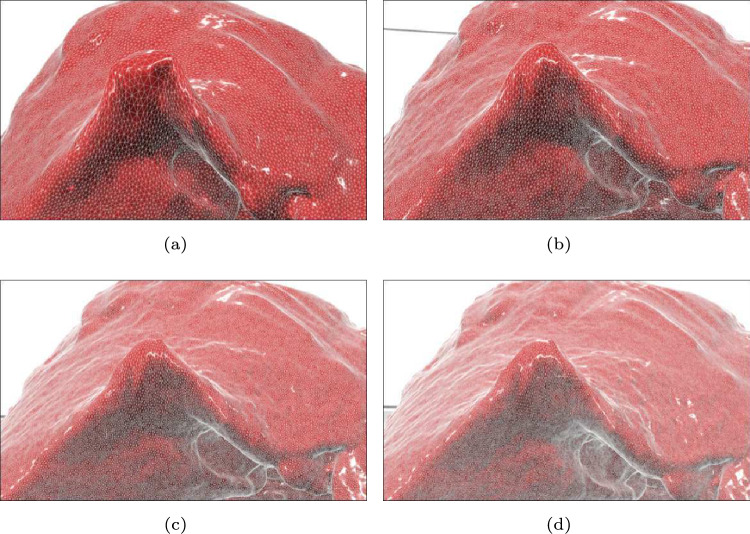
Screenshots of the deformation scene showing different mesh resolution. Mesh 1, Mesh 2, Mesh 3, and Mesh 4 correspond to (**a**), (**b**), (**c**), and (**d**), respectively

Figure 4 and Table 2 show the processing times $t_{\textrm{2}}$, $t_{\textrm{3c}}$, $t_{\textrm{4c}}$, and $t_{\textrm{4g}}$ for four meshes (Mesh 1 to Mesh 4) when $n_{\textrm{c}} = 1817$ in Case 3. Among them, only $t_{\textrm{3c}}$ increased linearly with $n_{\textrm{v}}$, while the other times remain nearly constant because the CPU-based deformation mapping is applied to each model vertex. Additionally, the nearly constant value of $t_{\textrm{4c}}$ suggested that the rendering performance of modern standard GPUs is sufficient for processing the models used in this study. Furthermore, Fig. 5 shows visual examples of the liver model deformation at different mesh resolutions.

**Fig. 6:**
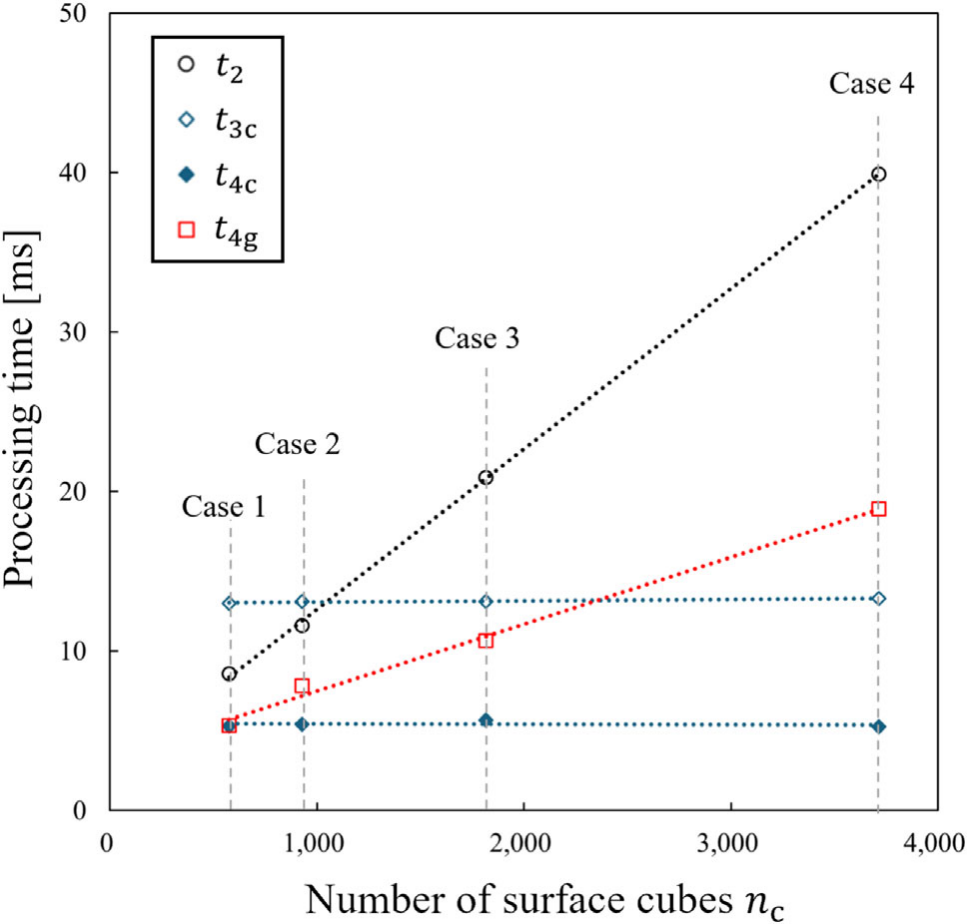
Variation of processing times with respect to $n_{\textrm{c}}$ in Experiment 2

**Fig. 7:**
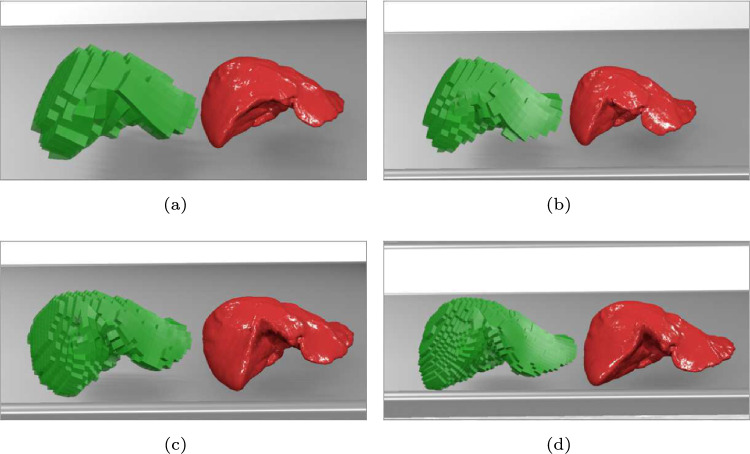
Renderings of the liver model with different cube resolutions. Case 1, Case 2, Case 3, and Case 4 correspond to (**a**), (**b**), (**c**), and (**d**), respectively

Figure 6 and Table 3 show the processing times $t_{\textrm{2}}$, $t_{\textrm{3c}}$, $t_{\textrm{4c}}$, and $t_{\textrm{4g}}$ for four cube cases (Case 1 to Case 4) when $n_{\textrm{v}} = 256,904$ in Mesh 4. In this case, $t_{\textrm{2}}$ and $t_{\textrm{4g}}$ increased linearly with $n_{\textrm{c}}$, while $t_{\textrm{3c}}$ and $t_{\textrm{4c}}$ remained almost constant. One reason for the increasement of $t_{\textrm{4g}}$ (in contrast to $t_{\textrm{4c}}$ remained constant) is that the eight vertex coordinates required for shader processing must be transmitted to the GPU for each cube in GPU-based deformation mapping. Figure 7 shows renderings of the liver model with different cube resolutions.

**Table 3:** Processing time values measured for four cube cases in Experiment 2

	$n_{\textrm{c}}$	$t_{\textrm{2}}$ [ms]	CPU-based			GPU-based	
			$t_{\textrm{3c}}$ [ms]	$t_{\textrm{4c}}$ [ms]	$T_{\textrm{c}}$ [ms]	$t_{\textrm{4g}}$ [ms]	$T_{\textrm{g}}$ [ms]
Case 1	576	8.6	13.0	5.3	26.9	5.3	13.9
Case 2	929	11.6	13.1	5.4	30.1	7.8	19.4
Case 3	1817	20.9	13.1	5.7	39.7	10.6	31.5
Case 4	3715	39.9	13.3	5.3	58.5	18.9	58.8

## Discussion

The experimental results in Chapter 4 provide a condition for determining which implementation is faster. If the number of model vertices $n_{\textrm{v}}$ is relatively large compared to the number of cubes $n_{\textrm{c}}$, Step 3 can be accelerated by the GPU’s vertex shader. The condition that the GPU-based implementation is faster for the same frame time is

11 $$\begin{aligned} t_{\textrm{4g}} < t_{\textrm{3c}} + t_{\textrm{4c}}. \end{aligned}$$

Figures 4 and 5 show that all processing times follow a linear trend with respect to both $n_{\textrm{v}}$ and $n_{\textrm{c}}$. The linearity indicates that $t_{\textrm{3c}} + t_{\textrm{4c}}$ and $t_{\textrm{4g}}$ can be expressed by:

12 $$\begin{aligned} t_{\textrm{3c}} + t_{\textrm{4c}}&=a_{\textrm{c}} n_{\textrm{v}} + b_{\textrm{c}} n_{\textrm{c}} + c_{\textrm{c}}, \end{aligned}$$

13 $$\begin{aligned} \textrm{and}\ t_{\textrm{4g}}&=a_{\textrm{g}} n_{\textrm{v}} + b_{\textrm{g}} n_{\textrm{c}} + c_{\textrm{g}}. \end{aligned}$$

This linearity is independent of hardware and can be applied in general PC environments.

Here, we derive the regression plane based on the seven samples in Tables 2 and 3. The equations are

14 $$\begin{aligned} t_{\textrm{3c}} + t_{\textrm{4c}}&=(5.19 \times 10^{-5})n_{\textrm{v}} + (7.43 \times 10^{-5})n_{\textrm{c}}\nonumber \\&\quad + 5.18\ [\text {ms}] (R^2=0.992), \end{aligned}$$

15 $$\begin{aligned} \textrm{and}\ t_{\textrm{4g}}&= (1.47 \times 10^{-6})n_{\textrm{v}} + (4.19 \times 10^{-3})n_{\textrm{c}}\nonumber \\&\quad + 2.92\ [\text {ms}] (R^2=0.994). \end{aligned}$$

The values in parentheses represent the coefficient of determination from the multiple linear regression. The condition that the GPU-based implementation is faster is:

16 $$\begin{aligned} (4.12 \times 10^{-3})n_{\textrm{c}} < (5.04 \times 10^{-5})n_{\textrm{v}} + 2.26. \end{aligned}$$

For example, the GPU-based implementation is faster if $n_{\textrm{c}}$ is less than 3696 when $n_{\textrm{v}}=256,904$.

The above results can be used to determine the upper limit of $t_{\textrm{2}}$ that can be processed in real time. For real-time applications, reducing the processing time of Step 2 is essential, which includes not only the physical simulation of elastic deformation but also simplifying and accelerating collision handling based on application requirements. This makes it possible to set the parameters for the PBD method and other physical simulation environments.

Simply increasing the number of cubes for accuracy would lead to the breakdown of this method from the standpoint of computational complexity. However, in the case of manipulation-based local deformation, the number of cubes is increased only in regions of large deformation, thus reducing the overall increase in cubes. Resolution control can be performed not only by the degree of deformation of the model but also by various other indicators, such as whether or not the part is the target of surgery and the distance from the surgeon’s viewpoint.

In this study, we used octree, one of the voxel-based models instead of polygon-based models, as a variable resolution model. Since the deformation and rendering processes are independent for each cube element, the processing algorithm can be simplified as long as the consistency of the data structure for each cube is maintained. This point will be an advantage in applying the model to future cutting operations. In particular, cutting high-resolution organ models requires the reconstruction of mesh models with a large number of vertices. This can temporarily stall the simulation. In the proposed method, since the organ model is divided into cubic regions, only reconstruction of a mesh with a small number of vertices is required, and the mesh to be processed can be identified quickly by an octree search.

## Conclusions

In this study, we proposed a method for accelerating octree cube deformation mapping to polygon models using GPU vertex shaders. The experimental results showed that the GPU can accelerate the mapping process in high-resolution organ models with a large number of vertices. The next steps include dynamic modification of the octree cube structure according to its deformation state and realization of operations such as cutting the organ model.
